# EHMTI-0357. Perceptions and experiences of cluster headache among patients, general practitioners and neurologists in the north of england: a qualitative study

**DOI:** 10.1186/1129-2377-15-S1-J1

**Published:** 2014-09-18

**Authors:** F Ahmed, L Dikomitis, HW Zafar, P Goadsby, K Paemeliere

**Affiliations:** 1 Neurology, Hull and East Yorkshire Hospitals NHS Trust, Hull, UK; 2 Social Sciences, University of Hull, Hull, UK; 3 Clinical Neuroscience, Kings College, London, UK; 4 Neurology, Ghent University Hospital, Ghent, Belgium

## Background

Very few qualitative studies on cluster headache have been conducted. As a result we have little in-depth understanding of the perceptions and experiences of cluster headache patients and the health professionals who treat them. With this research we aim to rectify that gap.

## Aim

The overall objective of the project is to gain insight into the perceptions, experiences and understandings of cluster headache from the perspective of three key stakeholder groups: the cluster headache patients, GPs and neurologists. We present here the findings of our literature study and identify the emergent themes which we will use during the interview studies for this project.

## Method

A qualitative study using semi-structured interviews with the three participant groups. A systematic qualitative methodology is applied to the transcribed interviews.

## Result

This study runs from August 2014 to June 2015 and data collection is currently in process. Therefore all results are tentative. Early findings show that this study sheds new light on the following aspects: early detection and diagnosis; health education and promotion; effective treatment and management of cluster headaches.

## Conclusion

This study will identify the particular challenges for each stakeholder group—patients, general practitioners and neurologists—with respect to early detection of cluster headaches and ways in which treatment and management can be facilitated. We envisage this study will raise awareness about the importance of early diagnosis, health education and the need for effective treatment pathways for cluster headaches.

